# An Alternative Technique in the Treatment of Celiac Axis Stenosis Diagnosed During Pancreaticoduodenectomy

**DOI:** 10.1155/1998/85406

**Published:** 1998

**Authors:** Marcel C. C. Machado, Sonia Penteado, André L. Montagnini, Marcel A. C. Machado

**Affiliations:** From the Department of Surgery University of São Paulo Brazil

## Abstract

Celiac compression is usually a benign condition, but when surgery necessitates division of collaterals from the superior mesenteric artery, it may cause life-threatening celiac organ ischemia. Celiac axis obstruction is found in 12.5% to 49.7% of patients during abdominal angiography. In such patients, the arterial blood supply to the stomach, spleen, and liver is sustained through extraordinarily welldeveloped pathways in the pancreas.

Though collateral pathways may be sacrificed during pancreaticoduodenectomy, only a small proportion of patients develop hepatic, gastric and splenic ischemia during the procedure. If the appropriate angiographic studies have not been obtained before pancreatic resection, a test occlusion of the gastroduodenal artery, as recommended by Bull *et al*. [2], should precede its ligation. The hepatic arteries are palpated before and after the test occlusion. In the occasional patient in whom the pulse diminishes during occlusion or if there is evidence of upper abdominal visceral ischemia, revascularization of the celiac circulation may be required. Reestablishment of the celiac circulation may be accomplished by the use of a vein graft between the aorta and the celiac tributaries. This article describes an alternative technique for revascularization of the celiac circulation without the use of a venous graft.


*HPB Surgery*, 1998, Vol. 10, pp. 371–373
Reprints available directly from the publisher
Photocopying permitted by license only

© 1998 OPA (Overseas Publishers Association)
Amsterdam B.V. Published under license
under the Harwood Academic Publishers imprint,
part of The Gordon and Breach Publishing Group.
Printed in India.

### An Alternative Technique in the Treatment of Celiac Axis Stenosis Diagnosed During Pancreaticoduodenectomy

MARCEL C. C. MACHADO[a,*], SONIA PENTEADO[a], ANDRÉ L. MONTAGNINI[a] and MARCEL A. C. MACHADO[a]

[a]*From the Department of Surgery, University of São Paulo, Brazil*

*(Received 20 September 1996; In final form 20 December 1996)*

**Celiac compression is usually a benign condition, but when surgery necessitates division of collaterals from the superior mesenteric artery, it may cause life-threatening celiac organ ischemia. Celiac axis obstruction is found in 12.5% to 49.7% of patients during abdominal angiography. In such patients, the arterial blood supply to the stomach, spleen, and liver is sustained through extraordinarily well-developed pathways in the pancreas.**

**Though collateral pathways may be sacrificed during pancreaticoduodenectomy, only a small proportion of patients develop hepatic, gastric and splenic ischemia during the procedure. If the appropriate angiographic studies have not been obtained before pancreatic resection, a test occlusion of the gastroduodenal artery, as recommended by Bull *et al.* [2], should precede its ligation. The hepatic arteries are palpated before and after the test occlusion. In the occasional patient in whom the pulse diminishes during occlusion or if there is evidence of upper abdominal visceral ischemia, revascularization of the celiac circulation may be required. Reestablishment of the celiac circulation may be accomplished by the use of a vein graft between the aorta and the celiac tributaries. This article describes an alternative technique for revascularization of the celiac circulation without the use of a venous graft.**

*Keywords:* Pancreaticoduodenectomy, surgery, celiac artery

### INTRODUCTION

Celiac axis obstruction is found in 12.5% to 49.7% of patients during abdominal angiography [1]. In such patients, the arterial blood supply to the stomach, spleen, and liver is sustained through extraordinarily well-developed pathways in the pancreas.

Though collateral pathways may be sacrificed during pancreaticoduodenectomy, only a small proportion of patients develop hepatic, gastric and splenic ischemia during the procedure. If the appropriate angiographic studies have not been obtained before pancreatic resection, a test occlusion of the gastroduodenal artery, as recommended by Bull *et al.* [2], should precede its ligation. The hepatic arteries are palpated before and after the test occlusion. In the occasional patient in whom the pulse diminishes

*Corresponding author: MARCEL A.C. MACHADO, A1. Casa Branca, 438#101 01408-001 São Paulo-SP Brazil. Telephone: 55-11-2530670, Facsimile: 55-11-2530670, e-mail : mmautran@br.homeshopping.com.br.

during occlusion or if there is evidence of upper abdominal visceral ischemia, revascularization of the celiac circulation may be required.

Reestablishment of the celiac circulation may be accomplished by the use of a vein graft between the aorta and the celiac tributaries.

This article describes an alternative technique for revascularization of the celiac circulation without the use of a venous graft.

### TECHNIQUE

Reestablishment of the celiac circulation is recommended to sustain normal blood flow to the celiac organs, especially when celiac flow is not detected after clamping the gastroduodenal artery or ischemia of celiac organs occurs after division of the gastroduodenal artery or pancreatic collaterals.

This technique provides revascularization of the celiac trunk by end-to-end anastomosis between the middle colic and gastroduodenal artery, since the middle colic artery in cases of celiac obstruction is enlarged enough to provide revascularization of the celiac organs. If after temporary occlusion of the middle colic artery no disturbance in the colonic circulation is seen, its ligation is performed. The artery is then isolated from the mesocolon with ligation of its branches with attention to preserve the main vascular arcade of the colon (Fig. 1). The middle colic artery is turned to the hepatic hilus and end-to-end anastomosed to the stump of gastroduodenal artery, providing vascularization to the hepatic bed and to the celiac axis (Fig. 2).

### DISCUSSION

Kohler *et al.* [3] described a patient with impairment of celiac circulation due to axis compression by the median arcuate ligament; this was successfully corrected by dividing the impinging diaphragmatic fibers, releasing the celiac artery. Bull *et al.* [2] reported two patients undergoing

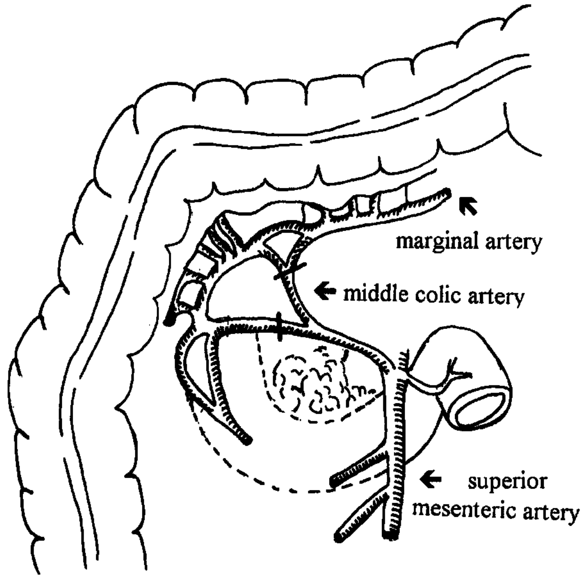

FIGURE 1 Preparation of middle colic artery for revascularization of the celiac circulation with preservation of marginal artery.

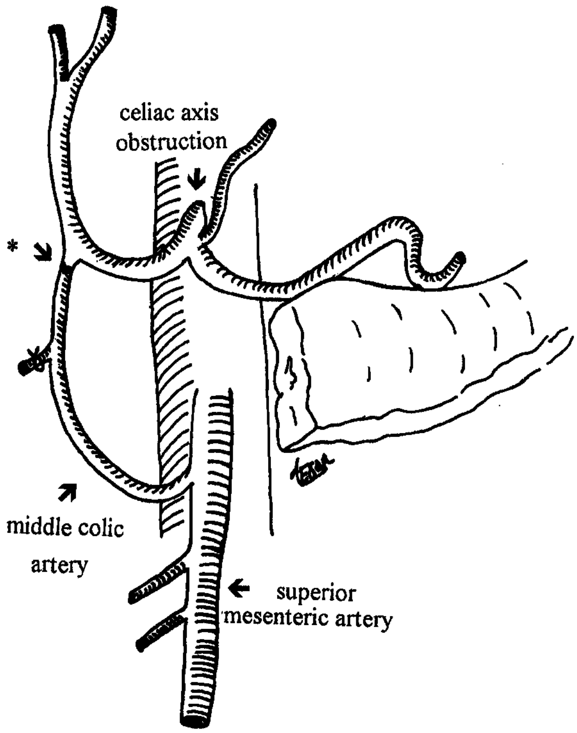

FIGURE 2 The middle colic artery is end-to-end anastomosed to the stump of gastroduodenal artery (*).

pancreaticoduodenectomy in whom obstruction of the celiac arterial circulation was not suspected until pancreatic resection had been achieved. Then, obvious ischemia of the liver, stomach, and pancreatic remnant led to discovery of celiac arterial occlusion, caused in both patients by compression and/or kinking of the axis by dense fibrotic tissue about its origin. Division of this periaoritic tissue relieved the obstruction, successfully restoring blood flow to the upper abdominal organs. Reestablishment of celiac circulation is need to avoid complications from celiac organ ischemia that may result in death. Peripancreatic inflammation in response either to ductal obstruction from ampullary carcinoma or especially from chronic pancreatitis may cause or contribute to the celiac obstruction.

However, most commonly, the lesions were atherosclerotic and, consequently, when requiring correction are best treated by revascularization of the celiac circulation. This may be accomplished by creating a bypass linking the aorta or superior mesenteric artery to the celiac system. But this technique involves a vein graft, usually an autograft of the saphenous vein.

Reestablishment of celiac circulation by creating an end-to-end anastomosis between middle colic and gastroduodenal arteries may be achieved without the use of an autograft. The mobility of the colon simplifies the anastomosis.

In our last 150 pancreaticoduodenectomies, we used this procedure in one patient with chronic pancreatitis in whom obstruction of the celiac axis was not suspected until pancreatic resection had been achieved. This obstruction was not detected upon gastroduodenal artery occlusion test. Immediately after pancreatectomy, the liver, stomach, pancreatic remnant and spleen were noted to be pale and obviously ischemic. After reestablishment of the celiac circulation, the liver, stomach and spleen promptly resumed their normal appearance. Postoperative duplex ultrasound indicated appropriate function of the graft and the patient remains well 15 months after the procedure.

The use of a middle colic artery seems to be a viable and simple option in reconstruction of the celiac circulation during pancreatectomy. However the mobilisation of the middle colic artery does carry a risk of colonic devascularisation even when care is taken to preserve the marginal artery of Drummond. This complication may be prevented by previous champing of middle colic artery and observation of the colonic circulation. In obese patients with thick mesocolon this procedure may be difficult or not suitable.

### *References*

[1] Miyata, M., Takao, T., Okuda, A., Sasako, Y. and Sunada, S. (1988). Pancreaticoduodenectomy for periampullary cancer associated with celiac occlusion: a case report: *Surgery*, **103**, 261–263.

[2] Bull, D. A., Hunter, G. C., Crabtree, T. G., Bernhard, V. M. and Putnam, C. W. (1993). Hepatic ischemia, caused by celiac axis compression, complicating pancreatico-duodenectomy. *Annals of Surg.*, **217**, 244–247.

[3] Kohler, T. R., Debas, H., Crames, M. and Strandness, D. E. (1990). Pancreaticoduodenectomy and the celiac compression syndrome. *Annals of Vascular Surgery*, **4**, 77–80.

### COMMENTARY

This paper describes a technique for revascularisation of the upper gastrointestinal tract in the presence of celiac axis obstruction. It may have a limited indication particularly in patients whose long saphenous vein has previously been removed. Additionally anastomosis of the vein graft to the supraceliac aorta can be extremely difficult due to limited access. Mobilisation of the middle colic artery does however carry risk of colonic devascularisation even where care is taken to preserve the marginal artery of Drammond. It would not be suitable for patients who are obese with a thick mesocolon.

Mr. I. F. Lane
Department of Vascular Surgery
University Hospital of Wales
Heath Park
CARDIFF
CF4 4XN

